# Efficacy and Safety of High-Intensity Interval Training in a Patient With Acute Lymphocytic Leukemia Receiving Consolidation Chemotherapy: A Case Report

**DOI:** 10.7759/cureus.84048

**Published:** 2025-05-13

**Authors:** Wataru Hanajima, Yuma Hirano, Natsuki Takeda, Tetsuyuki Nagafusa, Katsuya Yamauchi

**Affiliations:** 1 Department of Rehabilitation Medicine, Ayase Heart Rehabilitation Hospital, Tokyo, JPN; 2 Department of Rehabilitation Medicine, Hamamatsu University School of Medicine, Shizuoka, JPN

**Keywords:** acute lymphoblastic leukemia, consolidation chemotherapy, hematopoietic stem cell transplantation, high-intensity interval training, rehabilitation

## Abstract

Exercise tolerance prior to hematopoietic stem cell transplantation (HSCT) is an important prognostic indicator. Nevertheless, patients with acute lymphocytic leukemia (ALL) before HSCT have reduced exercise tolerance after multiple rounds of chemotherapy. Exercise is encouraged in these patients to prevent reduced exercise tolerance. However, exercise compliance is reduced during chemotherapy. Therefore, we focused on the potential role of high-intensity interval training (HIIT), which improves exercise tolerance but requires fewer interventions. The efficacy and safety of HIIT in patients undergoing chemotherapy remain unknown. Here, we report an exercise intervention that incorporated HIIT in a patient with ALL undergoing consolidation chemotherapy (CC). The patient was a 40-year-old female diagnosed with ALL for whom rehabilitation was implemented after initiation of induction chemotherapy. The patient underwent aerobic exercise on an ergometer (20 min at 50-60% intensity, using the Karvonen method) and weight-bearing resistance training (three sets of 10-20 repetitions at a 4-5 rating on the modified Borg scale) five times a week. The physical function assessment before the third CC session showed decreased exercise tolerance. An HIIT intervention was added as a more effective rehabilitation strategy for the patient. HIIT was performed on an ergometer. The protocol consisted of a 5-min warm-up, three to four sets of alternating between 80-90% peak oxygen uptake (peak VO_2_) and anaerobic threshold (AT) load for 3 min, and a 5-min cool-down. The number of HIIT interventions was nine of the 21 scheduled for rehabilitation. When HIIT was difficult, aerobic exercise was performed using an ergometer. Exercise tolerance improved without any adverse events, including bleeding, during rehabilitation. In conclusion, practicing HIIT sessions in patients receiving CC appears to safely enhance exercise tolerance with minimal intervention.

## Introduction

Acute lymphoblastic leukemia (ALL) is treated with hematopoietic stem cell transplantation (HSCT) as an additional therapy after a complete response is achieved. Before HSCT, patients with ALL receive multiple rounds of chemotherapy, including induction chemotherapy (IC) and consolidation chemotherapy (CC). The adverse effects of these treatments can lead to inactivity, thereby decreasing exercise tolerance [1]. Exercise tolerance in patients with ALL before HSCT is associated with complications and the survival rate after HSCT [2]. Therefore, patients with ALL must improve exercise tolerance during chemotherapy before undergoing HSCT. In general, combined aerobic and resistance training interventions are effective in preventing impaired exercise tolerance in patients with ALL [3]. However, leukemia chemotherapy targets total cell destruction, which causes severe side effects such as fatigue, pain, delayed hematopoiesis, and fever. These adverse effects reduce exercise compliance and impede improvements in exercise tolerance [4].

High-intensity interval training (HIIT) involves repetitions of high-intensity (80-90% of peak oxygen uptake (peak VO_2_)) and anaerobic threshold (AT) intensity exercise. This method can improve exercise tolerance in patients with cancer over a shorter period than conventional exercise therapy [5]. Therefore, HIIT may be effective for patients with low exercise compliance undergoing chemotherapy. HIIT has also been shown to be safe for patients undergoing chemotherapy [6].

Most studies examining the effects of HIIT have been performed in patients with colorectal cancer and chronic lymphocytic leukemia [7,8]. However, chemotherapy for patients with ALL targets the bone marrow than chemotherapy for other cancers, resulting in serious side effects such as bone marrow suppression. Therefore, during hospitalization, patients are isolated in clean rooms, the frequency of exercise participation decreases due to strong side effects, and physical function declines due to inactivity in the clean room. For patients with ALL who are about to undergo hematopoietic stem cell transplantation, the time until HSCT is short, so the importance of HIIT, which can be effective even with a small intervention, may be strong. Nevertheless, the effects of HIIT in patients with leukemia undergoing chemotherapy remain unknown. The feasibility and safety of HIIT have received limited attention. Here, we report an exercise intervention incorporating HIIT in a patient with ALL undergoing CC.

Interventions related to this case report were conducted in accordance with the Declaration of Helsinki for experiments involving humans. Written informed consent was obtained from the patient.

## Case presentation

The patient was a 40-year-old woman who had been experiencing fever, diarrhea, chest and back pain, and knee joint pain for 10 days prior to admission. A blood test revealed a white blood cell count of 10,440 cells/μL (37.0% blasts); she was diagnosed with ALL and admitted to the hospital on an emergency basis (at the time of diagnosis, she was 153 cm tall and weighed 52.7 kg). IC was immediately administered, and she achieved complete remission.

After two courses of CC (C1 and C2), the patient was temporarily discharged because her condition had stabilized, and she was subsequently readmitted for a third course of CC (C3). C3 was initiated 2 days after admission (day 0). 

At the time of readmission, the patient was receiving an anthracycline anticancer drug at a dose of 210 mg/m^2^ in doxorubicin equivalent; however, her left ventricular ejection fraction (LVEF) was 60.9%, indicating preserved cardiac function. Her forced vital capacity was 2.76 L. Her hemoglobin level was low at 9.8 g/dL due to side effects of the anticancer drug, and her platelet count was 30.1 × 10^4^/μL (Table 1).

**Table 1:** Changes in blood test results in Assessment 1 and Assessment 2 Na, sodium; K, potassium; CL, chlorine; BUN, Blood Urea Nitrogen; Cre, creatinine; T-Bil, Total Bilirubin; LD, lactate dehydrogenase; AST, aspartate aminotransferase; ALT, alanine aminotransferase; TP, total protein; Alb, albumin; CRP, C-Reactive Protein; WBC, White Blood Cell; RBC, Red Blood Cell; Hb, Hemoglobin; Hct, hematocrit; Plt, Platelet

	Assessment 1	Assessment 2	Reference range
Na (mmol/L)	136	140	138-145
K (mmol/L)	4.2	4.5	3.6-4.8
CL (mmol/L)	103	105	101-108
BUN (mg/dL)	8.4	8.7	8.0-20.0
Cre (mg/dL)	0.54	0.78	0.46-0.79
T-Bil (mg/dL)	0.51	0.34	0.4-1.5
LD (U/L)	288	762	124-222
AST (U/L)	16	28	13-30
ALT (U/L)	15	24	10-30
TP (g/dL)	6.3	6.1	6.6-8.1
Alb (g/dL)	4.2	4	4.1-5.1
CRP (mg/dL)	0.03	0.48	≦0.14
WBC (/μL)	3300	8340	3300-8600
RBC (×10^4^/μL)	334	245	386-492
Hb (mg/dL)	10.2	7.6	11.6-14.8
Hct (%)	32.3	23.1	35.1-44.4
Plt (×10^4^/μL)	30.1	6.6	15.8-34.8

The patient was independent in activities of daily living, with a Barthel Index of 100 points and a Performance Status of level 1 due to easy fatigability.

The initial physical function assessment (Assessment 1) was performed on the day before C3. Peak VO_2_ was calculated using cardiopulmonary exercise testing (CPET). The CPET was performed using a cycle ergometer (Aerobike 75XL III; Konami Sports & Life Co., Ltd., Tokyo, Japan) and an expiratory gas analyzer (Aeromonitor AE-310S; Minato Medical Science Co., Ltd., Osaka, Japan). The CPET protocol consisted of 5 min of rest, 4 min of 0 W warm-up, and a ramp load of 10 W per min until symptom limitation. AT was calculated using the V-slope method. The endpoints of CPET were difficulty in maintaining a rotational speed of 50 rpm, onset of chest symptoms, or when the patient reaches 90% of the predicted maximum heart rate, dangerous arrhythmia, or significant ST changes. Peak VO_2_ at Assessment 1 was 14.8 mL/kg/min, which was below the criterion for survival after HSCT. Thus, the patient's exercise tolerance was reduced [3].

The endpoint of the CPET was difficulty in maintaining a rotational speed of 50 rpm. Her AT was 9.1 mL/kg/min; the ventilation versus carbon dioxide production (VE vs VCO_2_) slope, which reflects cardiopulmonary function, was 31.2; the minimum ventilation/carbon dioxide production (minimum VE/VCO_2_) was 34.7 mL/mL; the peak respiratory quotient (peak R) was 1.4, peak heart rate (peak HR) was 154 bpm. The peak R was ≥1.1, indicating that the patient sustained exercise up to maximum load. Grip strength was 17.9 kg on the right side and 18.2 kg on the left side, and the 30-seconds chair stand test (CS-30) was performed 20 times. From the results of Assessment 1, muscle weakness was identified as a cause of decreased exercise tolerance.

During IC, the patient experienced pain in the chest, back, and flank; therefore, oxycodone 10 mg/day was administered, and rehabilitation was initiated. The pain decreased during C1 and C2, but nausea occurred as a side effect of chemotherapy. Therefore, metoclopramide was administered. Rehabilitation consisted of aerobic exercise using an ergometer (20 min at 50-60% intensity, using the Karvonen method) and weight-bearing resistance training (three sets of 10-20 repetitions at a 4-5 rating on the modified Borg scale), five times a week according to the patient's condition. However, the peak VO_2_ was low at assessment 1. HIIT was added, as it indicated the need for enhanced rehabilitation.

**Figure 1:**
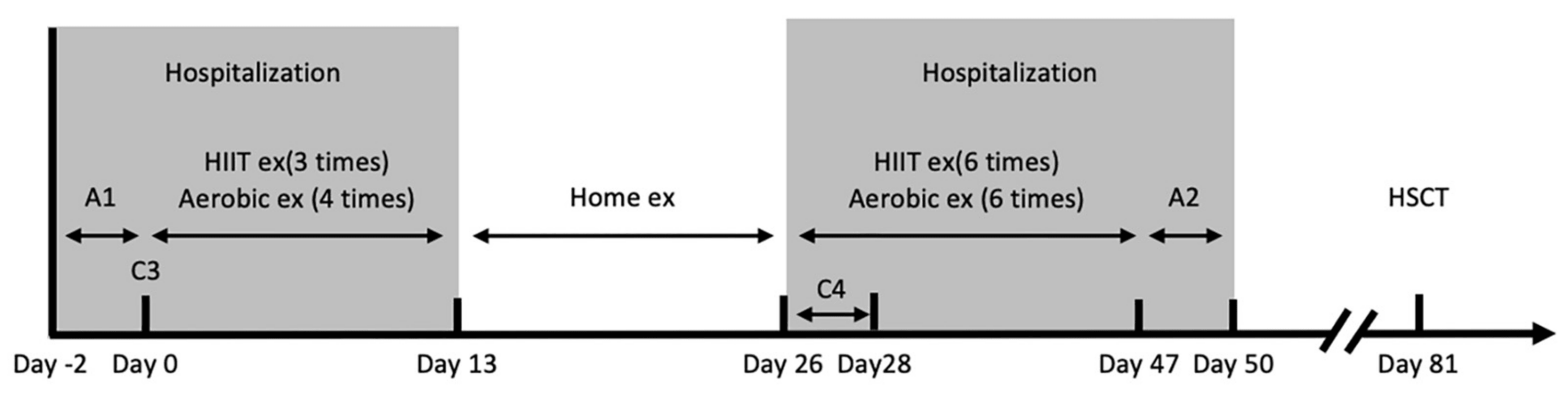
Clinical transitions and the number of rehabilitation interventions An initial physical function assessment (A1) was performed on the day before the third course of consolidation therapy (C3). After C3, a fourth course of consolidation therapy (C4) was administered after temporary discharge. HIIT was performed three times during C3 and six times during C4 for a total of nine sessions. The patient was reassessed for physical function on day 47 (A2) and was discharged on day 50. HSCT, hematopoietic stem cell transplant; HIIT, high-intensity interval training

**Figure 2:**
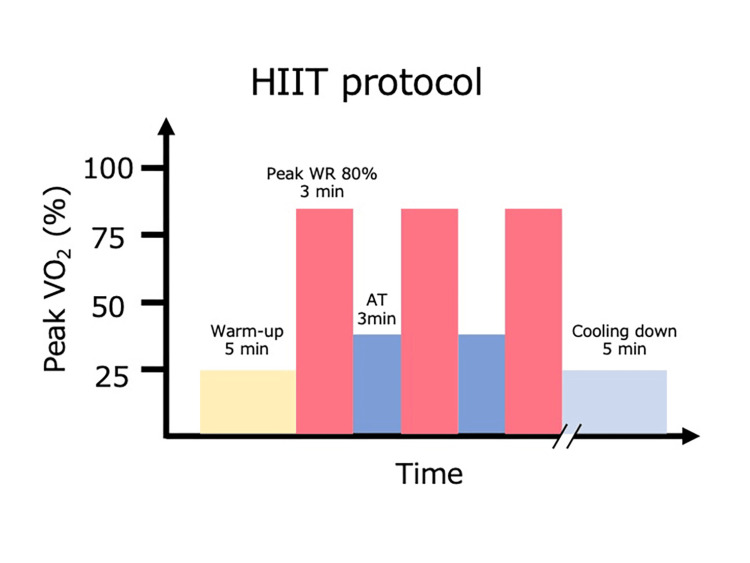
HIIT protocol Warm-up was performed for 5 min, followed by 3–4 sets of 3-min intervals of peak VO_2_ 80–90% and 3 min of AT. Cooling down was performed for 5 min. AT, anaerobic threshold; HIIT, high-intensity interval training; peak VO_2,_ peak oxygen uptake; peak WR, peak work rate

The patient’s clinical course is shown in Figure 1. Rehabilitation intervention consisted of HIIT, stretching, and resistance training. HIIT was performed on an ergometer. The protocol consisted of a 5-min warm-up, three to four sets of alternating between 80-90% peak VO_2_ and AT load for 3 min, and a 5-min cool-down (Figure 2) [9]. The resistance training was performed unsupervised. The number of exercise sessions was adjusted to maintain the patient’s subjective fatigue level between 4 and 5 on the modified Borg Scale. The participant was instructed to perform squats and calf raises. When HIIT was difficult, aerobic exercise was performed using an ergometer. The intensity was measured using the Karvonen method at 30-60% for 20 min.

The patient was discharged on day 13. At discharge, she was instructed to perform resistance training at the same intensity as during hospitalization and to walk 7000 steps/day. The patient was readmitted on day 27, and rehabilitation was resumed. The patient underwent a fourth course of CC (C4) beginning on day 31. The patient was reassessed for physical function on day 47 (Assessment 2) and discharged on day 50.

During the intervention period, the patient was administered chemotherapy, including oral dasatinib hydrate and prednisolone. In addition, antibacterial, antifungal, and antiviral agents were administered as concomitant medications. Aprepitant and acetaminophen were also administered to improve nausea, fever, and pain.

The patient's laboratory findings at Assessment 2 were as follows: LVEF, 62.2%; forced vital capacity, 2.86 L; hemoglobin, 7.6 g/dL; and platelet count, 6.6 ×10^4^/μL. In addition, activities of daily living scored 100 points on the Barthel Index, and the Performance Status was level 1; both remained unchanged. Peak VO_2_ improved from 14.8 to 16.1 mL/kg/min. The reason for terminating CPET was difficulty in maintaining a rotational speed of 50 rpm. AT increased from 9.1-9.8 mL/kg/min, VE vs. VCO_2 _slope, which reflected cardiopulmonary function, increased from 31.2-32.7; and the minimum VE/VCO_2_ decreased from 34.7 to 32.9. The peak respiratory quotient (peak R) was 1.5, and the peak heart rate (peak HR) was 166 bpm. Grip strength increased from 17.9 to 18.5 kg on the right side and decreased from 18.2 kg to 17.9 kg on the left side. The CS-30 improved from 20 to 26 times (Table 2).

**Table 2:** Changes in physical function in Assessment 1 and Assessment 2 Peak VO_2_, Peak oxygen uptake; AT, Anaerobic threshold; Resting VO_2_, resting oxygen uptake; Minimum VE/VCO_2_, the minimum ventilation/carbon dioxide production; VE vs VCO_2_ slope, ventilation versus carbon dioxide production slope; Peak VO_2_/HR, peak oxygen uptake/heart rate; Peak HR, Peak heart rate Peak; R, peak respiratory quotient; CS-30, 30-seconds chair stand test; LVEF, left ventricular ejection fraction; FVC, forced vital capacity

	Assessment 1	Assessment 2
Peak VO_2 _(mL/min/kg)	14.8	16.1
AT (mL/min/kg)	9.1	9.8
Resting VO_2_ (mL/min/kg)	114.0	112.0
Minimum VE/VCO_2 _	34.7	32.9
VE/VCO_2_ slope	31.2	32.7
Peak VO_2_/HR (mL/bpm)	5.2	5.0
Peak HR (bpm)	154.0	166.0
Peak R	1.4	1.5
Right-hand grip strength (kg)	17.9	18.5
Left-hand grip strength (kg)	18.2	17.9
CS-30 (times)	20.0	26.0
Body weight (kg)	52.7	50.7
LVEF (%)	60.9	62.2
FVC (L)	2.8	2.9

The number of HIIT interventions was nine of the 21 scheduled for rehabilitation. HIIT was not performed due to grade 1 fatigue, pain, and grade 3 platelet loss, according to the Common Terminology Criteria for Adverse Events v5.0. Alternatively, aerobic exercise was performed 10 times. Two of the 21 scheduled rehabilitation sessions were not performed due to grade 2 nausea or grade 3 anemia. The HIIT compliance rate ((completed HIIT sessions / planned HIIT sessions) × 100) was 42.9%, and the exercise compliance rate, including days, switched to aerobic exercise ((completed training sessions / planned training sessions) × 100) was 90.4%.

## Discussion

We describe a patient who received HIIT interventions for ALL rehabilitation during CC. To our knowledge, this is the first case report demonstrating the efficacy of HIIT exercises in a patient with ALL undergoing chemotherapy. The patient’s peak VO_2 _improved to more than 16.0 mL/kg/min, which was the standard criterion for survival before HSCT [3]. No adverse events associated with bleeding or low platelet counts were observed. The rate at which the patients underwent HIIT exercise was low (42.9%). However, when the number of aerobic exercise days was included, the compliance rate was 90.4%. These results suggest that HIIT is clinically feasible.

Effectiveness of exercise

Poor exercise tolerance before HIIT has been attributed to the side effects of chemotherapy, bed rest due to bone pain from ALL, prolonged hospitalization, and activity restrictions due to clean room treatment [10]. Because of these factors, the patient had a low peak VO_2_ of 14.8 mL/kg/min before HIIT.

Aerobic exercise at 50-75% of cardiac reserve intensity for patients with acute myeloid leukemia during chemotherapy has been reported to improve peak VO_2_ by 0.5 mL/kg/min after a 5-week intervention of 5 days per week [11] with patients increasing 1.3 mL/kg/min over a similar intervention period. This suggests that HIIT may be more effective than aerobic exercise for improving exercise tolerance. 

Furthermore, a similar study involving patients with CLL who underwent HIIT for 12 weeks at a load of 80-90% of peak VO_2_ reported a 5.3% increase in peak VO_2_. This patient experienced an 8.8% increase after 5 weeks of intervention, which is a shorter duration of improvement than reported in previous studies. This is thought to be due to the low baseline peak VO_2_ caused by chemotherapy.

Peak VO_2_ improvement with HIIT has been associated with skeletal muscle adaptations [8,12]. The improvement in peak VO_2_ was associated with increased muscle strength and enhanced skeletal muscle oxygen utilization. Patients undergoing chemotherapy exhibit increased inflammation, which decreases skeletal muscle mass and muscle strength [13]. Skeletal muscle mass was not measured, but there may have been minimal loss of skeletal muscle. In our patient, the skeletal muscle mass was not measured. However, resting VO_2_, a measure of the basal metabolic rate, was maintained, which suggests that skeletal muscle mass was maintained. In addition, the CS-30, a measure of lower extremity muscle strength, improved. This suggests that the anti-inflammatory effects of resistance training and HIIT were effective in maintaining skeletal muscle mass and improving muscle strength [14]. Hemoglobin is a factor that defines exercise tolerance related to oxygen delivery [15]. The patient exhibited a decrease in hemoglobin of 2.2 g/dL, which predicted a decrease in oxygen delivery. Decreased oxygen delivery causes skeletal muscles to become easily fatigued. However, the peak VO_2_ increased. HIIT improves mitochondrial function with fewer interventions than aerobic exercise [16]. Improvement in mitochondrial function indicates improved oxygen availability in skeletal muscles. This may have improved the peak VO_2_ despite reduced oxygen delivery.

Adherence to exercise programs and exercise safety

Compliance with aerobic exercise during chemotherapy has been reported to be 68.3% (range: 45.8-95.0%) [17]. The frequency with which our patient underwent HIIT was low (42.9%). However, when the number of aerobic exercise days was included, the compliance rate was 90.4%. This figure is similar to that of previous studies [17]. Factors that inhibit exercise include fatigue, nausea, and pain [18], which are the same factors that prevent HIIT from being performed.

One concern with exercise therapy in patients with ALL during chemotherapy is the risk of bleeding associated with low platelet counts. Exercise for patients receiving chemotherapy with low platelet counts is generally performed with appropriate adjustments made to exercise intensity according to platelet counts [19]. In our patient, HIIT was performed with platelet counts >3.1 × 10^4^/μLbased on previous studies. No adverse events associated with exercise, such as bleeding, were observed.

Patients may safely perform HIIT when the intervention is limited and chemotherapy side effects are minimal. We suggest that HIIT be performed as an alternative to aerobic exercise only when the patient is in good general condition. This approach may improve peak VO_2_ more safely and effectively than aerobic exercise.

A disadvantage of HIIT is that, when unsupervised, patients tend to exercise at lower intensities than indicated [20]. However, pre-transplant patients with ALL are under inpatient care and can exercise under supervision, enabling them to maintain appropriate intensity. Therefore, the disadvantages of HIIT are expected to be minimal.

This report has several limitations. The first is that, as a case report, no statistical analysis was performed. Therefore, when applying this HIIT intervention in clinical practice, the patient's condition and safety should be carefully considered. In addition, the intervention combined HIIT with resistance training and aerobic exercise. Therefore, the effect of HIIT alone remains unknown, and a larger study is needed to demonstrate its effect.

## Conclusions

We experienced a patient with ALL who practiced HIIT while undergoing CC. Peak VO_2_ improved despite limited interventions during CC. Additionally, no adverse events related to HIIT were observed, and it was safe to perform. Performing HIIT as an alternative to aerobic exercise during periods of minimal side effects may serve as a rehabilitation strategy for patients with ALL undergoing chemotherapy.
